# Case report: Systemic muscle involvement as the primary clinical manifestation of chronic active Epstein–Barr virus infection: A case-based review

**DOI:** 10.3389/fimmu.2022.1027859

**Published:** 2022-10-07

**Authors:** Shanfen Shi, Liangda Li, Cuiping Pan, Yandi Yang, Gun Chen, Yongping He

**Affiliations:** ^1^ Department of Rheumatology, The Affiliated People’s Hospital of Ningbo University, Ningbo, China; ^2^ Department of Neurology, The Affiliated People’s Hospital of Ningbo University, Ningbo, China; ^3^ Department of Pathology, The Affiliated People’s Hospital of Ningbo University, Ningbo, China

**Keywords:** chronic active Epstein–Barr virus infection, generalized myositis, polymyositis, lymphoma, CAEBV

## Abstract

Chronic active Epstein–Barr virus infection (CAEBV) is common in Asian countries and characterized by recurrent or persistent infectious mononucleosis-like symptoms. Here, we describe a rare case of CAEBV-associated generalized myositis with extranodal NK/T-cell lymphoma, who initially presented with swelling and muscle soreness in the extremities and was diagnosed as polymyositis at the initial stage. CAEBV-associated generalized myositis is different from polymyositis and other types of myositis. Furthermore, it is prone to lymphoma with poor prognosis.

## Introduction

Chronic active Epstein–Barr virus EBV infection (CAEBV) is a rare disease of progressive lymphocyte proliferation associated with chronic activation of EBV. Its incidence is closely related to elevated EBV DNA levels and EBV-positive lymphocytes infiltrating organs, which occur more often in children and young adults. Adults are less common and have a worse prognosis (1). Studies show that the median age of CAEBV is 19 years in the United States, and there is a lower median age in Asia at about 11.3 years. Its clinical manifestations vary by region: Lymphadenopathy and splenomegaly are the most common in the United States, whereas fever and hepatitis are the most common in Asia. Some patients can progress to life-threatening complications, such as hemophagocytic syndrome, interstitial pneumonia, and malignant lymphoma (2–4). However, CAEBV-related generalized myositis with systemic muscle involvement as the main manifestation is very rare and mainly reported in case reports, has a very poor prognosis, and lacks specific treatment measures (5). CAEBV-related generalized myositis disease is easy to misdiagnose as idiopathic myositis (polymyositis or dermatomyositis) in the early stage, but the pathogenesis, treatment response, and prognosis of the two are completely different. Here, we report one patient with a CAEBV-associated generalized myositis complicated with extranodal NK/T cell lymphoma. There is only one case report of CAEBV-associated generalized myositis combined with T cell lymphoma, whereas cases combined with extranodal NK/T cell lymphoma have not been reported yet. This paper aims to strengthen clinicians’ further understanding of such diseases through the report of this patient.

## Case report

**Figure 1:**
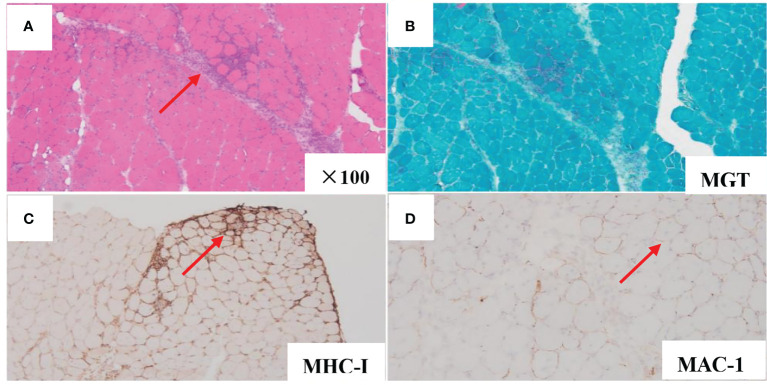
Muscle biopsy pathology analysis demonstrated chronic inflammatory cell infiltration in striated muscle tissue (HE stains, original magnification×100) **(A)**. MGT **(B)**. MHC-I (small peribundle fiber upregulation) **(C)**. MAC-1 (non-necrotic fiber membrane deposition) **(D)**.

**Figure 2:**
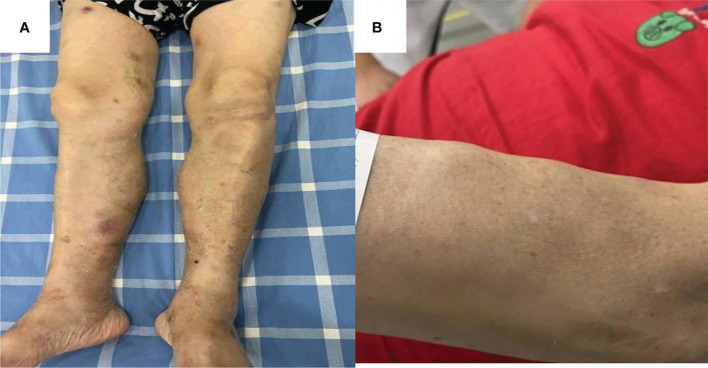
Subcutaneous limb nodules of both lower limbs **(A)**. Subcutaneous limb nodules of right upper limb **(B)**.

**Figure 3:**
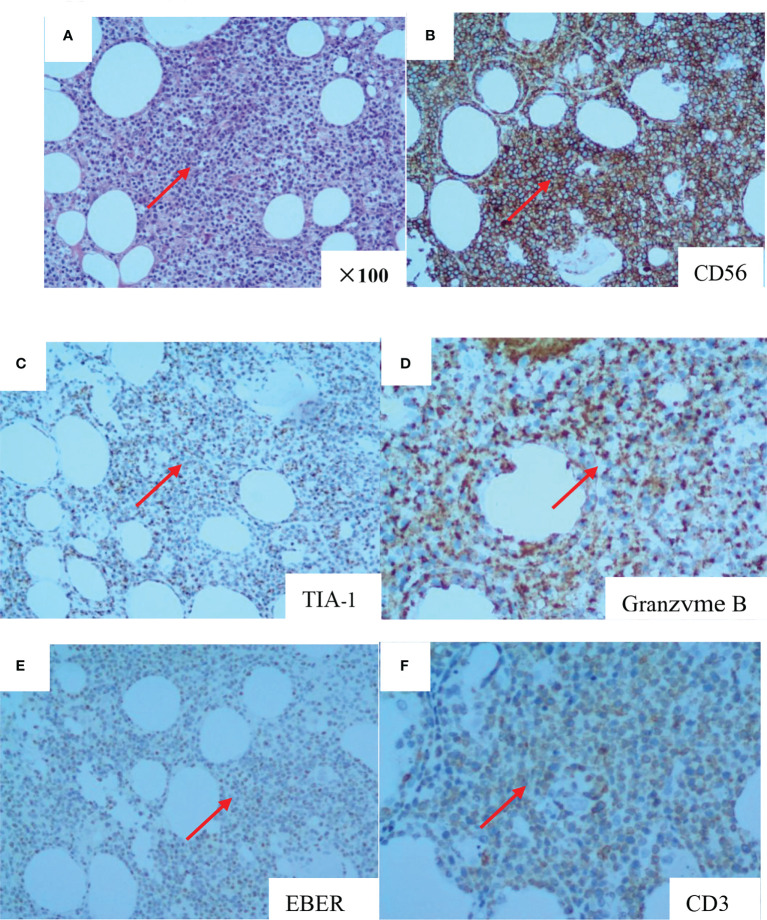
The pathology of subcutaneous nodule biopsy suggested diffuse infiltration of heterogeneous lymphoid cells in adipose tissue (HE stains, original magnifcation×100) **(A)**. CD56 (diffuse and strong +) **(B)**. TIA-1 (+) **(C)**. Granzyme B (+) **(D)**. EBER (partial +) **(E)**. CD3 (+) **(F)**.

A 66-year-old woman was admitted to our hospital in August 2020 due to “low fever, limb muscle soreness, weakness, and slowly progressive weight loss (about 10 kg). Laboratory tests showed that that the blood routine was basically normal, creatine kinase (CK): 847U/L, lactate dehydrogenase (LDH): 386 U/L, and EBV-IgG and IgA were positive in peripheral blood. EB virus DNA was 7.43*10^3^ copies/mL, and 16 myositis profile antibodies (including JO-1) were negative. MRI and electromyography tests indicated myositis. The detailed laboratory and auxiliary findings are shown in **Table 1**. Therefore, the diagnosis of this patient was (1) polymyositis and (2) EBV infection. After glucocorticoids, tacrolimus immunotherapy, and ganciclovir antiviral therapy, the patient’s body temperature returned to normal, but muscle pain persisted. Later, the myalgia was aggravated by glucocorticoid reduction, and the symptoms improved after glucocorticoid addition (40 mg) and replacement of the immunosuppressant (cyclophosphamide). After 9 months, the patient developed fever again, generalized muscle pain, and new skin erythema of the lower extremities. The laboratory tests showed CK 444 U/L and LDH 402 U/L. The peripheral serum EBV DNA was 5.88*10^5^ copies/mL, EBV-T DNA was 1.20*10^5^ copies/mL, EBV-B DNA was 4.66*10^4^ copies/mL, EBV-NK DNA was 1.73*10^5^ copies/mL. Further PET-CT showed systemic myositis with inflammatory hyperplasia of lymph nodes in the right neck, bilateral axilla, and bilateral inguinal areas. Bone marrow routine and biopsy showed no obvious abnormality. Muscle biopsy showed that the patient had striated muscle tissue with chronic inflammatory cell infiltration, and immunohistochemistry confirmed inflammatory myopathy, and the immunophenotypes were CD4(-), CD8(-), CD68(-), CD20 (weakly positive), MxA(-), MAC(+), p62(-), Dysferlin(+), R-Dystrophin(+) (**Figure 1**). Skin biopsy confirmed that the patient’s lesions were consistent with EBV(+) lymphoproliferative disease. According to the categorization of CAEBV: the case belonged to CAEBV infection involving skin expression (A2-A3). At this point, the patient was clearly diagnosed with CAEBV-associated generalized myositis infection. After the patient refused allogeneic hematopoietic stem cell transplantation, high-dose glucocorticoid (40 mg) combined with thalidomide (100 mg) therapy was given, and the patient’s symptoms were relieved, but the symptoms were still repeated. Unfortunately, after 4 months, the patient developed general weakness (especially in both lower extremities), and multiple subcutaneous painful nodules (maximum 4*3 cm) of different sizes developed all over the body with clear borders and different textures of hardness and softness (**Figure 2**). Further subcutaneous nodule pathological biopsy was confirmed as extranodal NK/T cell lymphoma (nasal type), and the immunophenotype was CD20(-), CD3(+), CD5(-), CD10(-), CD79a(-), CD43(+), Ki-67 (80%+), CD56 (diffuse strong +), EBER (partial +), GranzymeB(+), TIA-1(+), P53 (partial +), CD2(+), CD4(-), CD7(+), CD8(-), CD21(-), CD23(-), Bcl-2(-), Bcl-6(-), and C-Myc (20%+) (**Figure 3**). The patient’s peripheral serum EBV DNA was 5.88*10^5^ copies/mL, EBV-T DNA was 1.20*10^5^ copies/mL, EBV-B DNA was 4.66*10^4^ copies/mL, and EBV-NK DNA was 1.73*10^5^ copies/mL. Therefore, the final diagnosis of the patient was CAEBV infection–related generalized myositis combined with extranodal NK/T cell lymphoma (nasal type). P-GEMOX (gemcitabine 1.1 d1, oxaliplatin 130 mg d1, peaspargase 3750 U d2) regimen chemotherapy was given once, and PD-1 was added later, but the nodule progressively enlarged with severe pain, and the patient died within 4 months.

**Table 1:** The clinical characteristics and treatment of patients with three hospitalizations.

	First hospitalization	Second hospitalization	Third hospitalization
Symptom	low fever, limb muscle soreness, weakness, slowly progressive weight loss	fever again, generalized muscle pain, and new skin erythema of the lower extremities	general weakness and multiple subcutaneous painful nodules of different sizes developed all over the body
Laboratory test	CRP 9.6mg/L; GPT: 60U/L, GOT: 48U/L,CK: 847U/L, LDH: 386U/L	CRP 62.4mg/L, CK 444U/L, LDH 402U/L	CRP: 19.1mg/L, CK: 125 U/L, LDH 471U/L
EBV	peripheral blood EBV-IgG, IgA: +; EBV DNA 7.43×103copies/ml	EBV DNA 3.13×10^3^copies/mL	peripheral blood EBV DNA 5.88×10^5^copies/mL; EBV-T DNA 1.20×10^5^copies/mL; EBV-B DNA 4.66×10^4^copies/mL; EBV-NK DNA 1.73×10^5^copies/mL
Film degree exam	MRI indicated myositis	PET-CT showed systemic myositis with inflammatory hyperplasia of lymph nodes in the right neck, bilateral axilla, and bilateral inguinal areas	
Pathology		Muscle biopsy showed that the patient had striated muscle tissue with chronic inflammatory cell infiltration, immunohistochemistry confirmed inflammatory myopathy. Skin biopsy confirmed that the patient’s lesions were consistent with EBV(+) lymphoproliferative disease.	subcutaneous nodule pathological biopsy confirmed as extranodal NK/T cell lymphoma (nasal type).
Diagnosis	1. Polymyositis, 2. EB virus infection	CAEBV infection involving skin expression (A2-A3).	CAEBV infection–related generalized myositis, combined with extranodal NK/T cell lymphoma (nasal type)
Therapy	glucocorticoids, tacrolimus, ganciclovir	Glucocorticoid, thalidomide	P-GEMOX^1^, PD-1
Clinical status	Temperature returned to normal, but muscle pain persisted	Symptoms were relieved	Dead

1:gemcitabine 1.1 d1, oxaliplatin 130mg d1, peaspargase 3750U d2.

## Discussion

EBV virus is a double-stranded DNA herpes virus. More than 99% of normal people has been infected with EBV and persisted for life in the form of asymptomatic infection (6). Very few patients have primary infection or EBV reactivation, which develops into an incurable disease. Some of these patients develop fulminant mononucleosis and die within days or weeks of onset. Some develop a chronic course with persistent or intermittent infectious mononucleosis-like symptoms and tissue infiltration by EBV-positive T, NK, or lesser B cells (7). This chronic course of EBV infection was first proposed by Irelizier et al. in 1978, and was defined as CAEBV disease (8). The clinical manifestations are usually fever, persistent lymphadenopathy, splenomegaly, and EBV hepatitis.

Internationally, clinicians believe that the diagnosis of CAEBV is currently defined by the following criteria: (1) infectious mononucleosis-like disease persists for more than 3 months; (2) elevated EBV DNA titers in peripheral blood; (3) EBV infiltrated tissues of organs; (4) tissue infiltration of EBV viral protein and/or RNA; and (5) exclusion of underlying malignancy, autoimmune disease, or immunodeficiency (9, 10). Clinically, CAEBV often accumulates in the liver, spleen, and lymph nodes and presents an indolent clinical course, but once the disease progresses, it is life-threatening. At present, some scholars have proposed CAEBV with muscle infiltration as the main clinical manifestation based on case reports. Up to now, seven articles and nine cases of CAEBV with myositis as the main clinical manifestation have been reported, involving cardiac mechanism, eye muscle, skeletal muscle, etc. Only one patient was stable after rituximab treatment, and the rest died (5, 11–16). These cases suggest that CAEBV with myositis as the clinical manifestation has a worse prognosis. CAEBV with myositis as the main clinical manifestation is more rare, and it still lacks sufficient recognition and attention in clinical practice. It is often misdiagnosed as idiopathic myositis (polymyositis/dermatomyositis), but the prognosis is very different. Polymyositis is dominated by CD8+ T cells, whereas dermatomyositis is dominated by CD4+ T cells (17) and has a characteristic myositis antibody profile. However, in CAEBV patients, the clonal proliferation of EBV-infected cells is mainly CD4-, CD8- T cells (double negative T cells). CAEBV patients often die from fulminant infection, interstitial pneumonia, hemophagocytic syndrome, or progression to lymphoma. At present, only one case of CAEBV with systemic myositis as the clinical manifestation progressing to lymphoma was reported from Japanese scholars, and this patient progressed to T-cell lymphoma (5). The case we report here had fever, a common clinical symptom of CAEBV, and systemic myositis as the main clinical symptom. The difference, however, is that the patient we report had infiltrated T, B, and NK cells and eventually developed extranodal NK/T-cell lymphoma. This is unprecedented in previous case reports. The same as other case reports is that the patient in this case did not respond well to conventional hormones or antiviral and immunosuppressive agents. Even though this patient underwent chemotherapy and PD-1 immunotherapy in the lymphoma stage, it still failed to prevent the deterioration of the disease. This suggests that we should be highly vigilant in the clinical practice of patients with EBV activation.

Currently, there is no specific treatment for CAEBV-related generalized myositis, which may be closely related to its unknown etiology. CAEBV patients often have impaired T and NK cell activity (18, 19), and there are certain geographical differences. In Europe and the United States, EBV often infiltrates B cells, whereas in Southeast Asia, more T cells or NK cells infiltrate, and the prognosis is also worse (3, 4). Some scholars also suggest that the pathogenesis of CAEBV is related to gene mutation, and find that compound heterozygous mutation in perforin (20), MAGT1 mutation (21), and GATA2 mutation (22) may be involved in the occurrence of CAEBV. Somatic DDX3X mutation was found in NK or T cell infiltrating CAEBV (2). So far, no single gene mutation has been verified in CAEBV. Some scholars propose that allogeneic hematopoietic stem cell transplantation is the only way to cure CAEBV (23), but there is insufficient evidence. There are case reports of disease stabilized with rituximab, but adequate clinical support is also lacking (14). CAEBV-related generalized myositis is a clinically rare and intractable disease, and more clinical data and basic research are needed.

This paper reports the first case of CAEBV-related generalized myositis that progressed to extranodal NK/T cell lymphoma, suggesting that CAEBV-related generalized myositis is easily misdiagnosed as idiopathic myositis at first and can involve all T, NK, and B cells. It can rapidly progress to NT/T cell lymphoma and has an extremely poor prognosis. For such patients, early screening and early pathological biopsy are recommended, and physicians should be alert to the possibility of rapid progression to lymphoma. However, the report of this case also has limitations. Neither T nor NK cell activity was detected nor whole-genome sequencing performed, which failed to provide relevant information. It also suggests that we encounter such diseases, and gene sequencing may offer a deeper understanding of the disease.

## Data availability statement

The original contributions presented in the study are included in the article/supplementary material. Further inquiries can be directed to the corresponding author.

## Ethic statement

The studies involving human participants were reviewed and approved by the Affiliated People’s Hospital of Ningbo University’s Institutional Research Board. The patients/participants provided their written informed consent to participate in this study. Written informed consent was obtained from the individual(s) for the publication of any potentially identifiable images or data included in this article.

## Author contributions

SSF and PCP were the core physicians responsible for the patient care and drafted the manuscript. LLD contributed for the coordination of various departments involved. HYP helped the data collection for the article. GC contributed for the pathological part. YYD was principally responsible for the patient care and critically revised the manuscript. All the authors have approved the submitted version.

## Funding

This work was supported by the Science and Technology Project Yinzhou District Ningbo City (2021AS0054).

## Conflict of interest

The authors declare that the research was conducted in the absence of any commercial or financial relationships that could be construed as a potential conflict of interest.

## Publisher’s note

All claims expressed in this article are solely those of the authors and do not necessarily represent those of their affiliated organizations, or those of the publisher, the editors and the reviewers. Any product that may be evaluated in this article, or claim that may be made by its manufacturer, is not guaranteed or endorsed by the publisher.

## Abbreviations

CAEBV, Chronic active EBV infection; CK, creatine kinase; LDH, lactate dehydrogenase; HE, Hematoxylin-eosin; MGT, modified Gomori trichrome; MHC-1, major histo-compatibility-1; MAC-1, Macrophage-1 antigen; MRI, Magnetic Resonance Imaging; PET-CT, positron emission tomography/computed tomography; WBC, white blood cell; Hb, hemoglobin; PLT, Platelets; GPT, glutamic-pyruvic transaminase; GOT, glutamic-oxaloacetate transaminase; CRP, c-reactive protein.
